# Lanthanum enhances biomass and bioactive metabolite production in *Glycyrrhiza uralensis* via coordinated gene regulation

**DOI:** 10.3389/fpls.2025.1696545

**Published:** 2025-10-16

**Authors:** Yuanyang Shao, Yushi Wang, Yunhao Zhu, Lei Wang, Yashun Wang, Xingyu Guo, Enai Zhai, Huiqin Zou, Yonghong Yan

**Affiliations:** ^1^ School of Chinese Materia Medica, Beijing University of Chinese Medicine, Beijing, China; ^2^ Department of Chinese Medicine, The First Affiliated Hospital of Zhengzhou University, Zhengzhou, China; ^3^ School of Pharmacy, Henan University of Chinese Medicine, Zhengzhou, China; ^4^ Operating Room, Luoyang Orthopedic-Traumatological Hospital of Henan Province (Henan Provincial Orthopedic Hospital), Zhengzhou, China

**Keywords:** *Glycyrrhiza uralensis*, lanthanum, glycyrrhizic acid, liquiritin, liquiritigenin, transcriptome, metabolome

## Abstract

**Introduction:**

Glycyrrhizic acid, liquiritin, and liquiritigenin are key secondary metabolites in *Glycyrrhiza uralensis* Fisch. with significant pharmacological value. However, their relatively low content in the plant poses a challenge for efficient production. This study aimed to investigate the promotive effect and underlying mechanism of the rare earth element lanthanum (La) on the accumulation of these bioactive compounds.

**Methods:**

The impact of La treatment on *Glycyrrhiza uralensis* seedlings was systematically evaluated. Biomass parameters, including plant height, root fresh weight, and root dry weight, were measured. The contents of glycyrrhizic acid, liquiritin, liquiritigenin, and related metabolites were quantified using UPLC-MS/MS. The expression levels of key biosynthetic genes were analyzed via transcriptomic sequencing (RNA-seq). The distribution of La in plant tissues was determined by ICP-MS.

**Results:**

La treatment significantly promoted plant growth, increasing biomass parameters such as plant height, fresh weight, and dry weight. It also enhanced the accumulation of the target secondary metabolites, notably increasing the content of glycyrrhizic acid, liquiritin, and liquiritigenin. Transcriptome analysis revealed that La markedly upregulated the expression of key genes in the biosynthetic pathways: SQE and CYP88D6 in the glycyrrhizic acid pathway, and PAL, C4H, CHS, and 4CL in the liquiritin/liquiritigenin pathway. Significant positive correlations were observed among the La-induced increases in biomass, metabolite content, and the expression levels of these key genes. ICP-MS analysis confirmed substantial root-specific accumulation of La.

**Discussion:**

The results demonstrate that La enhances the accumulation of glycyrrhizic acid, liquiritin, and liquiritigenin via a dual mechanism that simultaneously increases biomass (thereby expanding the precursor pool) and upregulates the expression of key rate-limiting enzyme genes in their biosynthetic pathways. This synergistic action ultimately leads to elevated metabolite production. Our study thereby elucidates the mechanism by which La enhances bioactive metabolite yields in *Glycyrrhiza uralensis*, proposing a novel strategy for applying rare earth elements to improve the production of valuable compounds in medicinal plants.

## 1 Introduction

*Glycyrrhiza uralensis* Fisch is a renowned traditional herb valued for its functions in invigorating the spleen and replenishing qi, clearing heat and detoxifying, resolving phlegm and relieving cough, alleviating spasm and pain, and harmonizing the properties of other herbs (Li et al., 2024). Its key secondary metabolites, glycyrrhizic acid (Jiang et al., 2025), liquiritigenin (Hao et al., 2021)and liquiritin (Wu et al., 2025), are responsible for significant bioactive properties. Glycyrrhizic acid has demonstrated potential efficacy against various diseases, including SARS (Wu et al., 2025), COVID-19 (Le et al., 2024), and Alzheimer's disease (Song et al., 2025). Furthermore, liquiritin and liquiritigenin exhibit diverse pharmacological activities such as antitussive (He et al., 2019; Guo et al., 2024), anti-tumor (Wang et al., 2022; Lu et al., 2023), and neuroprotective (Lee et al., 2022; Sharma et al., 2023) effects. Considerable research efforts have been directed towards enhancing the accumulation of these crucial bioactive compounds in *Glycyrrhiza uralensis*. Strategies explored include microbial treatments utilizing bacteria (Sharma et al., 2023), fungi (Xie et al., 2023)and their extracts (Qiu et al., 2024), as well as non-microbial approaches involving the application of plant hormones (Li et al., 2020), radiation exposure (Zhang et al., 2018) and responses to drought (Li et al., 2023), salinity (Yao et al., 2024), and organic or chemical fertilizers (Cui et al., 2021; Shen et al., 2022). However, the practical implementation of these methods under field conditions often presents significant challenges, limiting their widespread adoption and application.

Rare earth elements (REEs) have been recognized as physiologically active substances in plants, playing a crucial role in regulating the accumulation of plant secondary metabolites. Studies on *Salvia miltiorrhiza* have demonstrated that La application elevates the levels of key tanshinones, such as tanshinone IIA and cryptotanshinone (Bian et al., 2016). Furthermore, research indicates that La at appropriate concentrations can enhance the accumulation of various bioactive compounds in *Glycyrrhiza uralensis*. For instance, La treatment has been shown to increase the content of flavonoids and polysaccharides in *Glycyrrhiza uralensis* seedlings (Pei et al., 2012) and stimulate the biosynthesis of glycyrrhizic acid in *Glycyrrhiza uralensis* cells (Liu et al., 2006). However, the promotive effects of La on the accumulation of the core secondary metabolites in *Glycyrrhiza uralensis*—namely glycyrrhizic acid, liquiritin, and liquiritigenin—and the underlying regulatory mechanisms remain unclear.

Metabolomics enables the quantitative analysis of small molecule metabolites in medicinal plants, facilitating the assessment of the overall metabolic responses to various treatments and elucidating their promotive effects on the accumulation of key secondary metabolites (Zhang et al., 2023). Transcriptomics, by profiling the global gene expression patterns under different conditions, provides crucial insights into the molecular mechanisms governing secondary metabolite biosynthesis (Lv et al., 2024). Inductively coupled plasma mass spectrometry (ICP-MS) offers precise determination of the distribution patterns of REEs within plant tissues, serving as a vital tool for deciphering the mode of action of REEs and their intrinsic mechanisms in modulating secondary metabolite content (D’Amore et al., 2023). The formation of secondary metabolites in medicinal plants is co-regulated by functional genes and environmental factors and relies on primary metabolites as essential precursors (Vogt, 2010). The level of primary metabolism directly influences biomass accumulation (e.g., root fresh/dry weight, plant height), and significant enhancement of biomass generally facilitates the synthesis of secondary metabolites (Petrova et al., 2024). The integrated application of multi-omics technologies—such as metabolomics, transcriptomics, and ionomics—demonstrates significant advantages in unraveling the mechanisms by which environmental factors regulate secondary metabolism (Zhou and Zheng, 2022). For instance, Yan et al. integrated transcriptomics, metabolomics, and physio-biochemical analyses to reveal substantial differentially expressed genes and differential metabolites in the biosynthesis pathways of phenylpropanoids, phospholipids, and nucleotides during the formation of nutritional traits in eggplant (*Solanum melongena*) *(* Yan et al., 2023). Yue et al. integrated metabolomic and transcriptomic profiling to reveal 11 key genes regulating flavonoid content variations in tea plants (*Camellia sinensis*) of different ages (Yue et al., 2022). Similarly, Li et al. employed combined transcriptomic and metabolomic approaches to discover that four RrMYB transcription factors are closely associated with the dynamic changes of secondary metabolites (including amino acids, phenolic acids, and flavonol derivatives) and antioxidant activity during the fruit development of *Rosa roxburghii* (Li et al., 2022). Collectively, these studies lay the groundwork for in-depth elucidation of how environmental factors (e.g., REE treatment) influence the accumulation of bioactive compounds in medicinal plants by modulating gene expression and the primary-secondary metabolic network.

Therefore, this study employs an integrated approach combining biomass analysis, targeted metabolomics, ICP-MS, and transcriptomics to systematically elucidate the regulatory mechanism of La on secondary metabolism in *Glycyrrhiza uralensis*: evaluating La-induced enhancement of primary metabolites (precursors for secondary metabolism); quantifying La-driven accumulation of key secondary metabolites (glycyrrhizic acid, liquiritin, liquiritigenin); mapping spatial correlations between La distribution and metabolite biosynthesis; and identifying key regulatory genes with their expression dynamics in secondary metabolic pathways. This work comprehensively deciphers the synergistic effects and molecular basis of La on *Glycyrrhiza uralensis* secondary metabolite accumulation, establishing a theoretical foundation for precision application of REEs in medicinal plant cultivation.

## 2 Materials and methods

### 2.1 Materials

*Glycyrrhiza uralensis* seeds were aseptically collected from Minqin County, Gansu Province, China. Seed scarification was performed by immersing the seeds in concentrated sulfuric acid (98%, ACS reagent grade) for 75 min with periodic agitation. Following acid treatment, the seeds were filtered through sterile gauze (200 mesh) and thoroughly rinsed under running deionized water until neutral pH was attained. Subsequently, the seeds were soaked in deionized water at room temperature (25 ± 2 °C) for 24 h, and water renewal was performed every 8 h (Dong et al., 2024). For germination trials, stratified seeds were aseptically transferred to sterilized potting mix (peat:vermiculite = 3:1 v/v) in standardized growth chambers maintained at 25 ± 1 °C with 16/8 h photoperiod (250 μmol m^-^² s^-^¹ PAR). Post-germination seedlings were cultivated under controlled conditions for 30 days prior to experimental treatments.

### 2.2 Sample treatment

In preliminary experiments, both foliar spray and root irrigation methods of La application were evaluated. Root irrigation with La solution resulted in visible leaf yellowing by day 9, indicating potential phytotoxicity to *Glycyrrhiza uralensis* seedlings. In contrast, seedlings subjected to foliar spray or treated with deionized water remained healthy. Therefore, foliar spraying was selected as the appropriate application method for subsequent formal experiments. Based on our preliminary concentration screening, which tested a range of LaCl_3_ solutions (50–500 mM), treatment with 100 mM LaCl_3_ was identified as the optimal concentration, as it resulted in the highest accumulation of key secondary metabolites—including glycyrrhizic acid, liquiritin, and liquiritigenin—while higher concentrations led to a decline in content, indicating a typical hormetic response. Accordingly, 30-day-old seedlings were foliar-sprayed with 100 mM L solution (≥99%, 10099-58-8, Macklin) using a handheld atomizer, while the control group (W) received equivalent volumes of deionized water. Harvested samples were triple-washed with ice-cold deionized water, flash-frozen in liquid nitrogen within 30 s of collection, and stored at -80 °C in pre-cooled cryogenic vials until further analysis.

### 2.3 Growth parameter measurements

Following a 7-day treatment period, specimens were gently washed with deionized water to remove surface soil. *Glycyrrhiza uralensis* plants were harvested and growth parameters assessed.

#### 2.3.1 Fresh weight determination

Fresh weights were determined immediately post-harvest by first washing whole plants with deionized water to remove soil, blotting surface moisture with filter paper, and measuring total fresh weight (FW) using an analytical balance (Sartorius QTX124IRU-1x, ± 0.1 mg) (Nishidono and Tanaka, 2025). Subsequently, shoots and roots were separated for partitioned FW measurements—shoot FW (aboveground tissues excluding senescent leaves) and root FW (soil-free, surface-dried underground tissues)—both weighed to 0.01 g accuracy.

#### 2.3.2 Dry weight determination

Separated tissues underwent dry weight (DW) determination through a standardized protocol. An initial 105 °C/15-min treatment deactivated enzymes. Subsequent drying occurred at tissue-specific temperatures: shoots at 50 °C and roots at 60 °C, both processed to constant weight. Final measurements used calibrated analytical balances with 0.01 g accuracy (Hou et al., 2018).

#### 2.3.3 Morphological measurements

Plant architecture was quantified using digital calipers (Mitutoyo NE30 531-103, 0.05 mm resolution). Plant height (integrated vertical extent from soil surface to apical meristem) was measured as the vertical distance from base to apical meristem, recorded to 0.1 cm accuracy with triplicate averaging (Jiang et al., 2025). Root length (distance from root crown to primary root tip) was determined from root crown to primary root tip (0.1 cm accuracy). Shoot height (vertical distance from base to apical meristem) was calculated as the difference between plant height and root length.

### 2.4 Metabolomic analysis of *Glycyrrhiza uralensis* induced by lanthanum

#### 2.4.1 Preparation of *Glycyrrhiza uralensis* samples

A 50% (v/v) methanol solution (analytical grade) was prepared for extraction. Cryopreserved *Glycyrrhiza uralensis* samples were thawed, accurately weighed, and homogenized with the methanol solution at a 1:10 (w/v) ratio using a planetary ball mill (800 rpm, 45 s grinding/10 s pause, 2 cycles) (Chen et al., 2023). The homogenate was centrifuged at 12,000 rpm for 30 min, and the supernatant was carefully collected through 0.22 μm filtration for subsequent analysis.

#### 2.4.2 Standard solution preparation

Certified reference standards of glycyrrhizic acid (purity ≥98%, Shanghai Yuanye), liquiritin (≥98%, Shanghai Yuanye), liquiritigenin (≥98%, Shanghai Yuanye), isoliquiritigenin (≥96%, Shanghai Yuanye), isoliquiritin (≥98%, Shanghai Yuanye), and glycyrrhetinic acid (≥98%, Shanghai Yuanye) were accurately weighed (0.00146 g, 0.00103 g, 0.00203 g, 0.00286 g, 0.00242 g, and 0.00160 g, respectively), dissolved in HPLC-grade methanol (Merck), and serially diluted to prepare stock solutions (1 mg/mL). Calibration curves were established by diluting the stock solutions to concentrations ranging from 0.1 to 100 μg/mL.

#### 2.4.3 Chromatographic conditions

Separation was performed on a Waters ACQUITY UPLC BEH C18 column (2.1 mm × 100 mm, 1.7 μm) maintained at 40 °C. The mobile phase consisted of (A) 0.1% (v/v) aqueous formic acid and (B) acetonitrile with 0.1% formic acid. A gradient elution program was applied as follows: 0.0–0.5 min, 1% B; 0.5–10.0 min, 1–95% B; 10.0–12.0 min, 95% B; 12.0–14.0 min, 95–5% B; 14.0–15.0 min, 5% B-1% B (Liu et al., 2025). The flow rate was 0.3 mL/min, and the injection volume was 5 μL. All samples were stored at 4 °C in the autosampler prior to analysis (Wang et al., 2024).

#### 2.4.4 Mass spectrometric conditions

Ionization was achieved using a heated electrospray ionization (HESI) source in negative ion mode. Key parameters were optimized as follows (Li et al., 2024): spray voltage, 3.2 kV; sheath gas flow rate, 35 arbitrary units (arb); auxiliary gas flow rate, 15 arbs; capillary temperature, 350 °C; probe heater temperature, 300 °C; S-lens RF level, 50%; and mass resolution, 70,000 (full width at half maximum, FWHM) at m/z 200. Full-scan data were acquired over a mass range of m/z 100–1500.

#### 2.4.5 Data processing

Raw data were preprocessed using Progenesis QI software (Nonlinear Dynamics, 2014, version 1.0) for noise reduction, peak alignment, and normalization. Multivariate analyses included unsupervised PCA (SIMCA-P 16.0) to assess clustering trends and supervised OPLS-DA (VIP >1.5) to identify discriminative metabolites. Differential metabolites were defined as VIP >1.5, |log_2_FC| >1, and *p* < 0.05 (FDR-adjusted) validated with reference standards.

### 2.5 ICP-MS determination of lanthanum in *Glycyrrhiza uralensis*

#### 2.5.1 Digestion of *Glycyrrhiza uralensis* samples

Precisely weighed triplicate root and leaf samples were transferred to nitric acid-preconditioned digestion tubes (20% HNO_3_ soak, 36 h; ultrapure water rinse). After securing tubes, 5 mL 68% HNO_3_ was added incrementally followed by 20-min room temperature equilibration. Samples were carefully transferred to a graphite digester and subjected to programmed heating in stages: first at 80 °C (10-min ramp, 10-min hold), then at 150 °C (10-min ramp, 10-min hold), and finally at 200 °C (10-min ramp, 40-min hold). Digestion proceeded at 200 °C until solutions clarified with complete residue dissolution. Tubes were cooled in a fume hood, then filtered into 10-mL volumetric flasks (Mazarakioti et al., 2022). Digests were diluted to final volume, homogenized by inversion, and stored for analysis. Parallel blank preparations followed identical procedures.

#### 2.5.2 Standard solution preparation

Working standards were prepared by serial dilution of a 100 mg/L stock solution using 1% nitric acid as the diluent, generating calibration standards at concentrations of 10, 20, 40, 60, 80, and 100 µg/L for curve establishment.

#### 2.5.3 ICP-MS instrumental conditions

Operating in helium collision mode with optimized parameters, the instrument was configured as follows: radiofrequency power 1,550 W; plasma gas flow 14 L/min; auxiliary gas flow 0.8 L/min; nebulizer gas flow 1.06 L/min; sampling depth 7.0 mm; peristaltic pump speed 50 rpm. Each sample underwent 30 s uptake and 20 s stabilization prior to triplicate measurements with 20 sweeps per replicate (Hur et al., 2023).

#### 2.5.4 Lanthanum quantitation methodology

Quantitation employed external calibration with matrix-matched lanthanum standards (10-100 μg/L in 1% HNO_3_, diluted from 100 mg/L stock). Sequentially analyzed *Glycyrrhiza uralensis* root and leaf digests alongside method blanks. Final concentrations were calculated via linear regression based on the calibration curve.

### 2.6 Transcriptomics study on *Glycyrrhiza uralensis* induced by lanthanum

#### 2.6.1 RNA-seq sample preparation

Total RNA was isolated from root tissues of *Glycyrrhiza uralensis* samples in both W and La groups using a plant-specific RNA extraction kit (CW3145S, Kangwei Century Biotechnology Co., Ltd., Beijing). Subsequently, the purity and quantity of the Total RNA were assessed using a NanoDrop 2000 spectrophotometer (Thermo Scientific, USA); its integrity was evaluated using an Agilent 2100 Bioanalyzer (Agilent Technologies, Santa Clara, CA, USA).

#### 2.6.2 cDNA library construction and sequencing

Qualified RNA samples were reverse-transcribed into double-stranded cDNA (ds cDNA). The ds cDNA was then purified, end-repaired, and ligated with sequencing adapters to construct the cDNA library. The constructed cDNA libraries were subjected to paired-end RNA sequencing on the Illumina NovaSeq 6000 platform, which was performed by Beijing Personal Novogene Co., Ltd.

#### 2.6.3 Sequence data processing and alignment

The *Glycyrrhiza uralensis* reference genome (Mochida et al., 2017) served as the alignment template. Raw paired-end sequencing reads underwent standard preprocessing using Fastp software (v0.23.2). This included adapter trimming, quality filtering (removing low-quality bases/reads), and base error correction to generate high-quality processed reads. Subsequently, these processed reads were aligned to the reference genome using the HISAT2 aligner (v2.2.1) with its default parameters. Alignment quality was assessed based on overall mapping rates.

#### 2.6.4 Gene expression quantification and differential expression analysis

Gene expression levels were quantified based on the reference genome annotation (Mochida et al., 2017). First, transcript abundance of protein-coding genes was determined by aligning sequencing reads to the reference genome using sequence similarity mapping. Read counts per gene were generated with HTSeq-count (v0.13.5) (Putri et al., 2022), and gene expression levels were calculated as FPKM (Fragments Per Kilobase Million). Differential expression analysis was performed using DESeq2 (v1.4.5). Genes with |log_2_ (fold change) | ≥ 1 and false discovery rate (FDR) < 0.05 were identified as differentially expressed genes (DEGs). Based on the sign of log_2_ (fold change):log_2_FC > 0 indicated up-regulated DEGs, log_2_FC < 0 indicated down-regulated DEGs.

#### 2.6.5 Functional enrichment analysis of DEGs

Gene Ontology (GO) and Kyoto Encyclopedia of Genes and Genomes (KEGG) pathway enrichment analyses were conducted for DEGs using a hypergeometric test. Terms with p-value < 0.05 were considered significantly enriched. The most enriched terms were selected as follows: GO analysis: Top 10 terms per category (Biological Process, Cellular Component, Molecular Function) where associated DEGs showed |log_2_FC| > 1 (fold change > 2), sorted by descending -log_10_(p-value). KEGG analysis: Top 20 pathways where associated DEGs showed |log_2_FC| > 1, sorted by descending -log_10_(p-value).

#### 2.6.6 qRT-PCR validation

Six randomly selected DEGs from W and La groups were validated via qRT-PCR to confirm RNA-seq accuracy. Using total RNA isolated from root tissues, cDNA was synthesized by reverse transcription with the PrimeScript™ RT Reagent Kit (Takara.RR037A, Takara Bio Dalian Co., Ltd., China). qPCR amplification used gene-specific primers (**Supplementary Table 1**) on a CFX96 Real-Time PCR System (Bio-Rad, USA). The actin gene served as internal control. Relative expression was calculated by the 2^−ΔΔCT^ method (Munshi et al., 2025), with statistical analysis in GraphPad Prism v9.5.0.

#### 2.6.7 Statistical analyses

All experiments were independently repeated at least three times, and the results are presented as mean ± standard deviation (SD). Statistical analyses were performed using SPSS (version 26.0; IBM Corp., Armonk, NY, USA) and GraphPad Prism (version 9.4.1; GraphPad Software, San Diego, CA, USA). Data normality and homogeneity of variances were assessed using the Shapiro-Wilk test and Levene's test, respectively. For datasets meeting both normality and homogeneity of variance assumptions, one-way analysis of variance (ANOVA) was conducted. Upon detection of significant differences (*p* < 0.05), *post hoc* pairwise comparisons were performed using Tukey's Honestly Significant Difference (HSD) test. This test controls the Family-wise Error Rate (FWER) to manage the risk of Type I error by identifying which specific group pairs exhibited significant differences. Bonferroni correction was applied where appropriate to adjust the significance level for multiple comparisons. This method operates by dividing the original significance level (α = 0.05) by the total number of comparisons, thereby adjusting the significance threshold (critical p-value) to control the FWER. For datasets with unequal variances (heteroscedasticity), Welch's t-test was employed for group comparisons. For non-normally distributed data, the non-parametric Mann-Whitney U test was used. Statistical significance is denoted as follows: *p < 0.05, **p < 0.01, and ***p < 0.001.

## 3 Results

### 3.1 Growth parameter responses to lanthanum treatment

**Figure 1:**
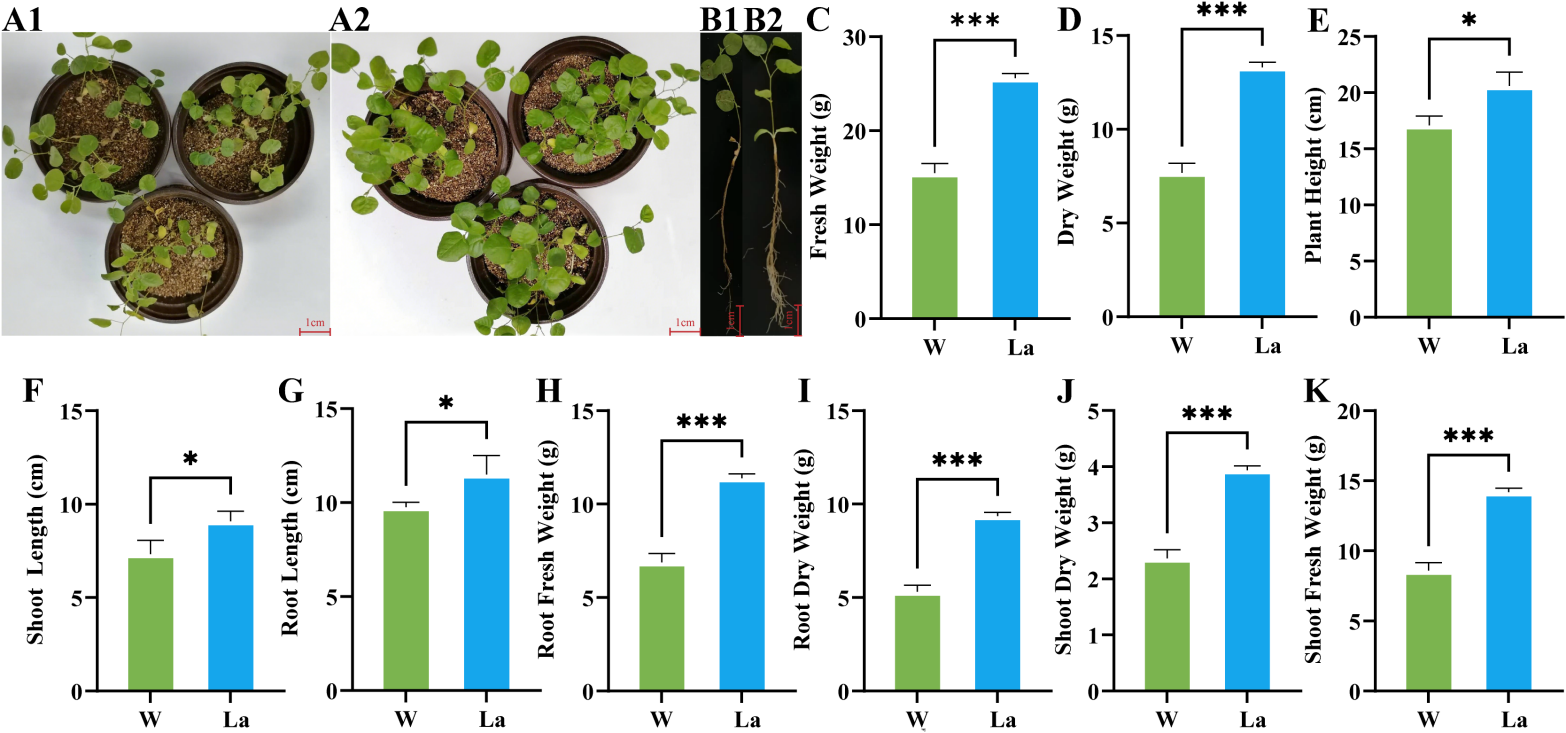
Effects of La treatment on growth parameters of *Glycyrrhiza uralensis* plant. **(A1)** *Glycyrrhiza uralensis* plant treated with W; **(A2)** *Glycyrrhiza uralensis* plant of La; **(B1)** *Glycyrrhiza uralensis* plant treated with W; **(B2)** *Glycyrrhiza uralensis* plant of La; **(C)** Fresh weight; **(D)** Dry weight; **(E)** Plant height; **(F)** Shoot height; **(G)**Root length; **(H)** Root fresh weight; **(I)** Root dry weight; **(J)** Shoot dry weight; **(K)** Shoot fresh weight. Data are presented as mean ± SD (n = 3). Significant differences between W and La groups were determined by Student's t-test (*p < 0.05, ***p < 0.001).

FW of *Glycyrrhiza uralensis* seedlings exhibited a highly significant increase in response to La treatment (**Figures 1A, B**). The total FW of La plants were 1.65 times greater than that of the control group, representing a significant increase of 65.32% (*p* < 0.001) (**Figure 1C**). This growth stimulation was observed in both aerial and subterranean tissues. The shoot FW (aboveground tissues excluding senescent leaves) increased by 63.48% (1.63 times control; *p* < 0.001) (**Figure 1K**), while the root FW (soil-free, surface-dried underground tissues) increased by 62.90% (1.63 times control; *p* < 0.001) (**Figure 1H**).

DW accumulation demonstrated an even more pronounced response to La. The total DW increased by 73.26% compared to the control, equivalent to 1.73 times the control value (*p* < 0.001) (**Figure 1D**). Partitioned analysis revealed a significant increase in shoot DW of 66.70% (1.67 times control; *p* < 0.001) (**Figure 1J**). The most substantial gain was observed in root DW, which increased by 76.18% (1.76 times control; *p* < 0.001) (**Figure 1I**).

La treatment significantly altered the morphology of *Glycyrrhiza uralensis* seedlings. Shoot length increased by 24.97% in La plants compared to controls (1.24 times control, *p* < 0.05) (**Figure 1F**). Root length showed a significant increase of 17.75% (1.17 times control, *p* < 0.05) (**Figure 1G**). Additionally, the plant height rose by 24.09% in La seedlings relative to the control group (1.24 times control, *p* < 0.05) (**Figure 1E**).

### 3.2 Targeted metabolomic analysis of major secondary metabolites in *Glycyrrhiza uralensis* in response to Lanthanum treatment

**Figure 2:**
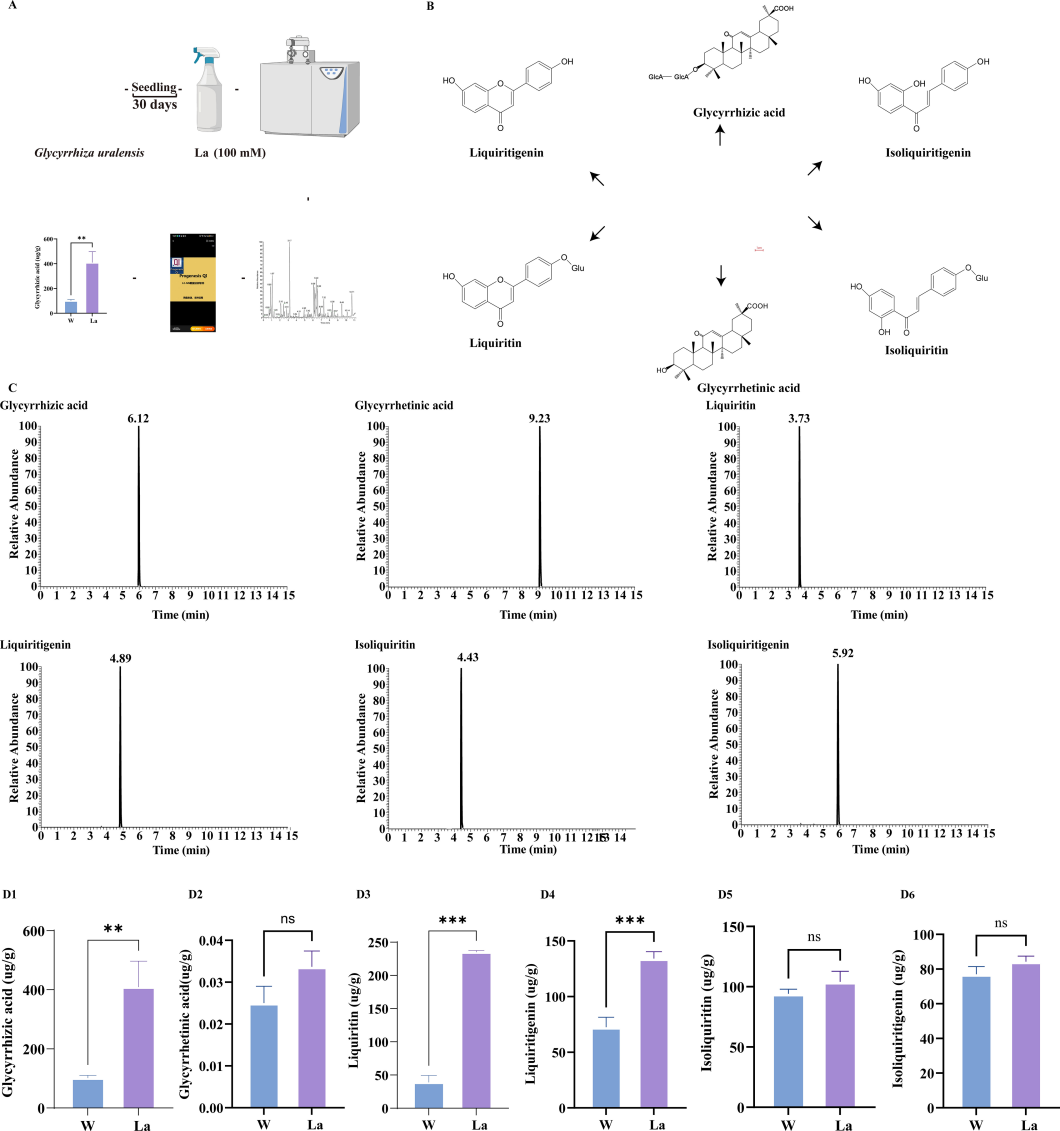
Metabolomics analysis of *Glycyrrhiza uralensis* secondary metabolites. **(A)** Metabolomics study design; **(B)** *Glycyrrhiza uralensis* and its major secondary metabolites; **(C)** Chromatograms of six constituents in *Glycyrrhiza uralensis* with internal standard; **(D)** Contents of six main secondary metabolites of *Glycyrrhiza uralensis* **(D1)**: Glycyrrhizic acid; **(D2)**: Glycyrrhetinic acid; **(D3)**: Liquiritin; **(D4)**: Liquiritigenin; **(D5)**: Isoliquiritin **(D6)**: Isoliquiritigenin). Data are presented as mean ± SD (n = 3). Significant differences between W and La groups were determined by Student's t-test (**p < 0.01, ***p < 0.001).

Glycyrrhizic acid, glycyrrhetinic acid, liquiritin, liquiritigenin, isoliquiritin, and isoliquiritigenin represent the primary bioactive constituents of *Glycyrrhiza uralensis* (**Figure 2B**). To investigate the impact of La treatment on these compounds, a targeted metabolomic analysis quantified their concentrations in *Glycyrrhiza uralensis* roots from both W and La groups (**Figure 2A, C**). All concentrations are expressed as mean ± SD on a fresh weight basis (μg/g FW). The concentrations of glycyrrhizic acid, liquiritigenin, and liquiritin were significantly higher in La roots compared to W (**Supplementary Table 2**). Glycyrrhizic acid was quantified at 268.72 ± 16.89 μg/g fresh weight (FW) in La roots, significantly higher than the 179.77 ± 11.53 μg/g FW observed in W roots, representing a 1.46-fold increase (*p* < 0.01). Liquiritigenin concentration reached 165.38 ± 19.24 μg/g FW under La treatment, markedly elevated compared to 28.39 ± 7.03 μg/g FW in W roots (a 5.82-fold increase, (*p* < 0.001)). Liquiritin levels were 11.30 ± 0.95 μg/g FW in the La group versus 5.00 ± 2.57 μg/g FW in W, showing a significant 2.26-fold upregulation (*p* < 0.001). La treatment induced 1.46-, 5.82-, and 2.26-fold increases in glycyrrhizic acid, liquiritigenin, and liquiritin content, respectively. In contrast, glycyrrhetinic acid, isoliquiritigenin, and isoliquiritin showed no statistically significant differences between groups (**Figure 2D**).

### 3.3 ICP-MS determination of lanthanum content in *Glycyrrhiza uralensis*

**Figure 3:**
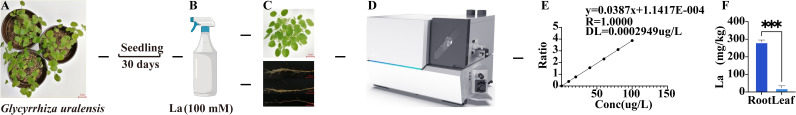
La content detection in *Glycyrrhiza uralensis* plants. **(A)** Initial Glycyrrhiza plant; **(B)** Foliar spray with La solution; **(C)** Harvested root and leaf samples; **(D)** La quantification by ICP-MS; **(E)** La standard calibration curve; **(F)** La content in root and leaf tissues. Data are presented as mean ± SD (n = 3). Significant differences between W and La groups were determined by Student's t-test (***p < 0.001).

ICP-MS was employed to quantify La concentrations in different tissues of *Glycyrrhiza uralensis* (**Figures 3A–E**). The results revealed substantial accumulation of La in-root tissues, reaching 283.36μg/g (dry weight). In contrast, La concentrations in leaf tissues were significantly lower, measuring only 22.10 μg/g (dry weight). Statistical analysis confirmed an extremely significant difference (*p* < 0.001) in La concentration between roots and leaves (**Figure 3F**). Further calculation indicated that the root La concentration was 11.81-fold higher than that in the leaves (**Supplementary Table 3**).

### 3.4 Transcriptomics study on *Glycyrrhiza uralensis* induced by lanthanum

#### 3.4.1 RNA quality assessment

Agarose gel electrophoresis confirmed intact RNA without degradation or contamination in all six samples (**Supplementary Figure 1**). Quantitative analysis revealed the following quality parameters: Control group samples exhibited RNA concentrations of 59 ng/μL (2.07 μg total), 32 ng/μL (1.12 μg), and 42 ng/μL (1.47 μg) for samples 1-3, respectively; with corresponding OD_260/280_/ OD_260/230_ ratios of 2.11/1.08, 2.10/0.78, and 2.17/0.97; 25S/18S rRNA ratios of 1.2, 1.3, and 0.9; and RIN values of 6.8, 7.5, and 7.0. La group samples showed concentrations of 39 ng/μL (1.37 μg total), 40 ng/μL (1.40 μg), and 36 ng/μL (1.26 μg) for samples 1-3, respectively; with OD_260/280_/OD_260/230_ ratios of 2.17/0.71, 2.12/0.68, and 2.13/0.67; 25S/18S rRNA ratios of 0.8, 0.8, and 0.9; and RIN values of 6.8, 6.8, and 7.0 (**Supplementary Table 4**, **Supplementary Figure S1**). Integration of electrophoretic integrity, spectrophotometric purity, fluorometric quantification, and bioanalyzer assessment confirmed that all RNA samples met quality thresholds for library construction and RNA sequencing.

#### 3.4.2 Transcriptomic profiling of *Glycyrrhiza uralensis* response to lanthanum treatment

##### 3.4.2.1 Sequencing data processing

**Figure 4:**
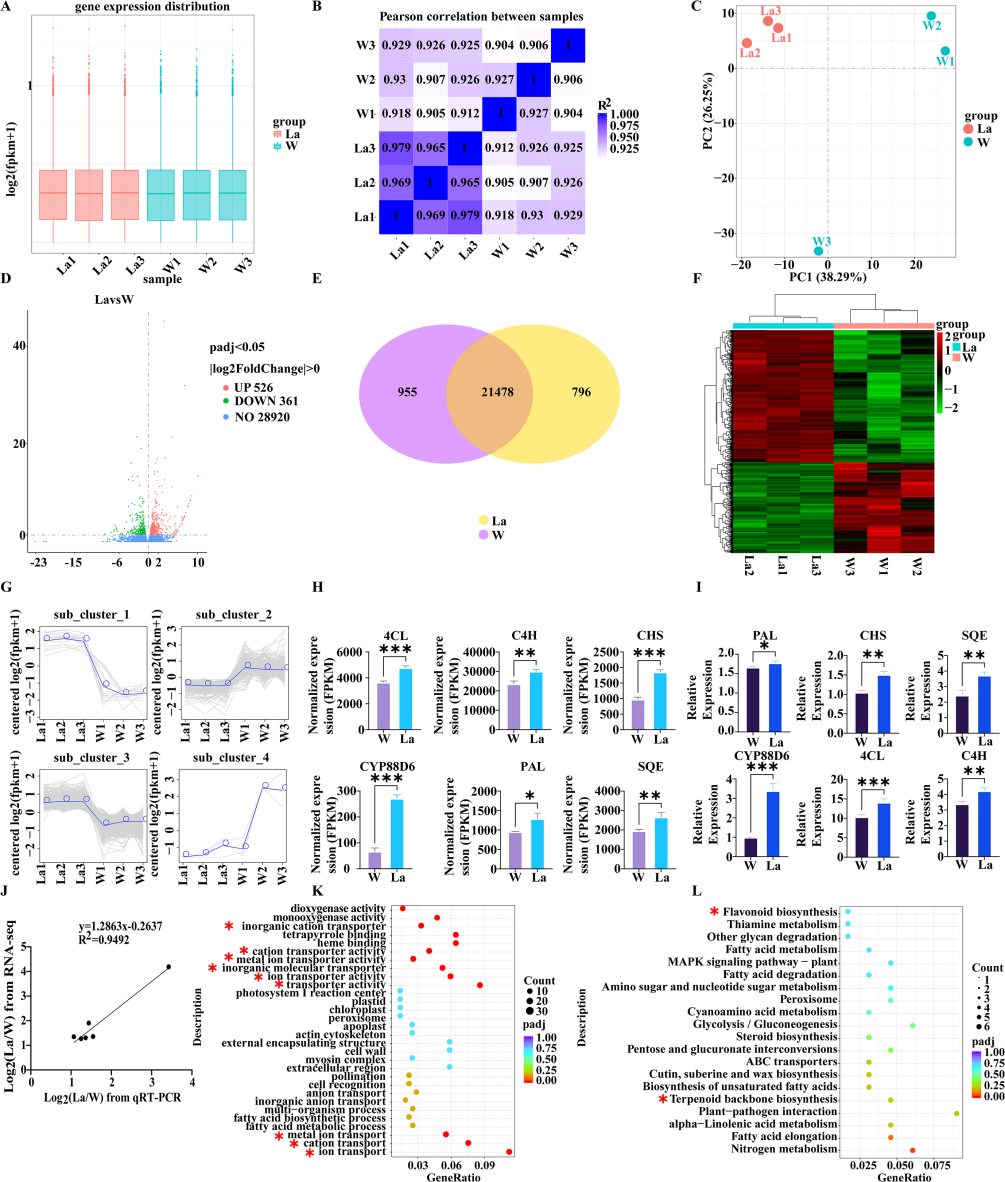
Transcriptomic Profiling of *Glycyrrhiza uralensis* in response to La treatment. **(A)** Gene expression distribution across samples; **(B)** Inter-sample correlation matrix; **(C)** PCA of transcriptomes; **(D)** Volcano plot of DEGs; **(E)** Venn diagram of overlapping DEGs between comparison groups; **(F)** Hierarchical clustering of DEGs; **(G)** Temporal expression trends of clustered DEGs; **(H)** FPKM distribution of six key DEGs; **(I)** qRT-PCR validation of six key DEGs; **(J)** Correlation of RNA-seq and qRT-PCR fold-changes; **(K)** GO enrichment of DEGs; **(L)** KEGG pathway enrichment. Data are presented as mean ± SD (n = 3). Significant differences between W and La groups were determined by Student's t-test (*p < 0.05, **p < 0.01, ***p < 0.001).

RNA sequencing generated approximately 267 million raw reads. After quality control filtering, 260 million high-quality clean reads were obtained, representing a filtering efficiency of 97.38%. On average, approximately 91.80% of the clean reads were successfully mapped to the *Glycyrrhiza uralensis* reference genome (Mochida et al., 2017), with 89.27% uniquely mapped (**Supplementary Table 5**). Gene expression levels, quantified as FPKM, were calculated for both the La group and the W group. Violin plots were employed to visualize the distribution and dispersion of gene expression levels between the two groups (**Figure 4A**). Principal Component Analysis (PCA) revealed good reproducibility among biological replicates within each group and a clear separation between the treatment groups, indicating significant differences in their transcriptomic profiles (**Figure 4C**). Pearson correlation analysis further confirmed strong correlations between samples within groups (**Figure 4B**).

#### 3.4.3 Differential expression analysis

Analysis of differentially expressed genes (DEGs) identified 887 DEGs between the two treatment groups. Compared to the control group, 526 genes were significantly up-regulated and 361 genes were significantly down-regulated in the La group (**Figures 4D-G**).

#### 3.4.4 Functional annotation and enrichment analysis

To elucidate the biological functions of the DEGs in *Glycyrrhiza uralensis* growth, functional annotation was performed using the GO database. A total of 1647 DEGs were annotated and enriched in 412 GO terms. Notably, 242 DEGs were significantly enriched in 12 specific GO terms. These significantly enriched terms were primarily associated with “inorganic cation transport” and “transporter activity” (**Figure 4K**). Annotation using the KEGG pathway database revealed that 103 DEGs were enriched in 54 pathways. Among these, 24 DEGs showed significant enrichment in 7 key pathways. These significantly enriched pathways included “Terpenoid backbone biosynthesis” (ath00900), “Flavonoid biosynthesis” (ath00941), “Carbon metabolism” (ath01200), Glycolysis/Gluconeogenesis” (ath00010), “Nitrogen metabolism” (ath00910), “Plant hormone signal transduction” (ath04075)” and “Plant-pathogen interaction” (ath04626) (**Figure 4L**).

Further screening of key DEGs revealed that, compared to the control, the expression levels of CHS and C4H were significantly up-regulated (*p* < 0.001 and *p* < 0.01, respectively), while the expression levels of SQE and CYP88D6 were significantly down-regulated (*p* < 0.01 and *p* < 0.001, respectively) in the La group (**Figure 4H**).

##### 3.4.5 qRT-PCR validation

To validate the reliability of the RNA-seq data, qRT-PCR analysis was performed on selected differentially expressed genes (DEGs). The log_2_ (fold change) values were compared between RNA-seq and qRT-PCR (bar plots, **Figure 4I**). Linear regression analysis demonstrated a strong correlation between the two methods (**Figure 4J**), confirming that the expression patterns detected by qRT-PCR were highly consistent with the RNA-seq results. This validates the robustness of the transcriptomic data.

### 3.5 Co-occurrence analysis among physiological growth parameters, metabolites and differential genes

**Figure 5:**
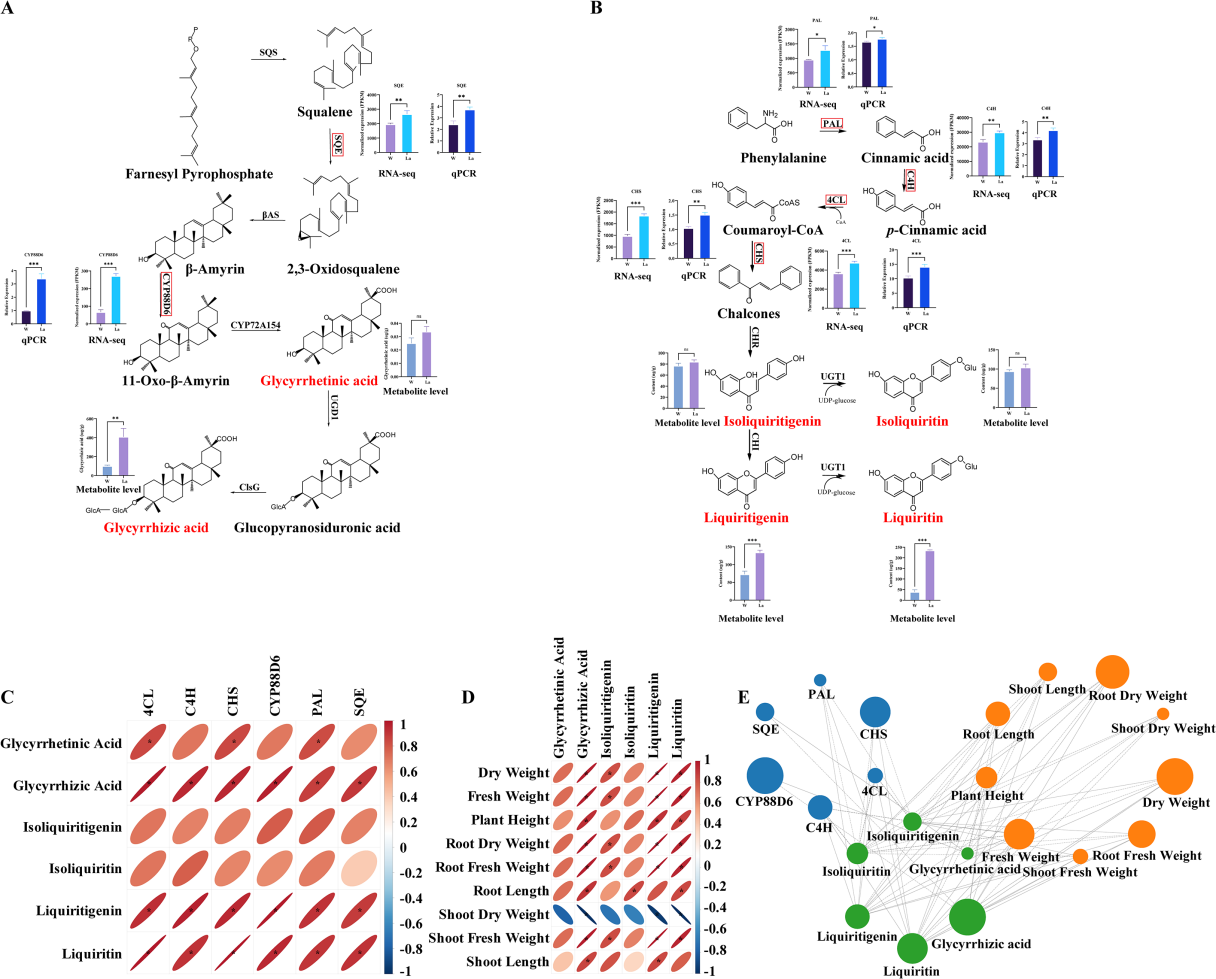
Integrated correlation network of bioactive metabolites, biosynthetic genes, and growth traits in *Glycyrrhiza uralensis* under La treatment. **(A)** Glycyrrhizic acid biosynthesis pathway;**(B)** Liquiritin, liquiritigenin, isoliquiritigenin, and isoliquiritin biosynthesis pathway; **(C)** Correlation analysis of bioactive metabolites and biosynthetic genes; **(D)** Correlation Analysis of Bioactive Metabolites and Growth Traits; **(E)** Co-occurrence network displaying the correlation values between physiological growth parameters, metabolites and differential genes. Size of the node indicates the degree of these variables. Solid lines indicate significant correlations (*p* < 0.05), while dashed lines denote non-significant correlations (*p* ≥ 0.05).

Pearson correlation analysis integrating targeted metabolomics (six major secondary metabolites), transcriptomics (six key regulatory genes), and physiological growth parameters (nine traits including fresh/dry weight, biomass partitioning, and morphological dimensions) in *Glycyrrhiza uralensis* seedlings revealed significant co-occurrence patterns (**Figures 5C–E**). Glycyrrhizic acid exhibited extremely strong positive correlations with CYP88D6 (r = 0.97, *p* < 0.01) and SQE (r = 0.90, *p* < 0.05) expression, a trend that was also visually consistent with the metabolic pathway depicted in **Figure 5A**. Liquiritin showed near-perfect positive correlations with 4CL (r = 0.99, *p* < 0.001) and CHS (r = 0.99, p < 0.001), alongside strong correlations with C4H (r = 0.92, *p* < 0.01) and *PAL* (r = 0.90, *p* < 0.05). Liquiritigenin demonstrated consistently strong positive correlations with PAL (r = 0.92, *p* < 0.05), 4CL (r = 0.93, *p* < 0.01), C4H (r = 0.96, *p* < 0.01), and CHS (r = 0.94, *p* < 0.01). These correlations are further supported by the pathway illustration in **Figure 5B**. Concomitantly, glycyrrhizic acid, liquiritin, and liquiritigenin concentrations were strongly positively correlated (r > 0.80, *p* < 0.05) with key biomass metrics: DW, FW, root dry weight (RDW), root fresh weight (RFW), and shoot fresh weight (SFW), as well as with plant height and root length. Isoliquiritin specifically correlated with root length (r > 0.84, *p* < 0.05), while isoliquiritigenin showed significant positive associations with DW, FW, RDW, RFW, and SFW (r > 0.80, *p* < 0.05).

## 4 Discussion

### 4.1 Lanthanum as a biochemical elicitor: targeted enhancement of key secondary metabolites in *Glycyrrhiza uralensis*

REEs, particularly La which functions as a “super calcium analogue” demonstrate significant potential for enhancing secondary metabolite biosynthesis across plant species, as evidenced by documented increases in specialized compounds in *Citrus sinensis (* Hanif et al., 2024), *Oryza sativa (* Chen and Zhong, 2024), and *Solanum nigrum (* Guo et al., 2024). This elicitation effect extends notably to *Glycyrrhiza uralensis*, where La application elevates cellular biomass concurrent with stimulated glycyrrhizic acid production (Liu et al., 2006). Field studies further quantify La's growth-promoting capacity, reporting 70.62% and 46.61% increases in aboveground and underground biomass yields respectively versus controls, alongside significant flavonoid accumulation (Pei et al., 2012). Mechanistic specificity is observed in anion-dependent responses: La (NO_3_)_3_ preferentially enhances glycyrrhizic acid and glycyrrhetinic acid synthesisw (Jia et al., 2024a), whereas LaCl_3_ promotes glycyrrhizic acid and liquiritin accumulation (Jia et al., 2024b). Our experimental data corroborate these patterns: La-treated *Glycyrrhiza uralensis* exhibited significantly elevated glycyrrhizic acid levels (*p* < 0.01), with even more pronounced increases in liquiritin and liquiritigenin (*p* < 0.001). Although non-significant upward trends occurred in glycyrrhetinic acid, isoliquiritigenin, and isoliquiritin, the statistically validated enhancement of core triterpenoid (glycyrrhizic acid) and key flavanone (liquiritin/liquiritigenin) metabolites confirms that optimal La concentrations act as a potent biochemical elicitor. This targeted metabolic induction aligns with La's established role in upregulating the expression and activity of key biosynthetic enzymes, including PAL, C4H, 4CL, and CHS in the phenylpropanoid pathway and SQE and CYP88D6 in the triterpenoid pathway, positioning REEs as promising agricultural tools for quality-driven cultivation of medicinal plants.

Furthermore, the role of La as an abiotic elicitor may extend beyond optimal growth conditions to enhance plant resilience under environmental stress. Notably, recent studies have demonstrated that La supplementation, particularly as La(NO_3_)_3_, can significantly fortify *Glycyrrhiza uralensis* against salt stress by interconnected physiological and molecular pathways (Jia et al., 2024a). This enhanced resilience is attributed to La-induced improvements in ionic homeostasis (e.g., reduced Na^+^ uptake), bolstered antioxidant capacity, and the concomitant upregulation of stress-responsive genes and secondary metabolic pathways—many of which overlap with the biosynthetic routes (e.g., phenylpropanoid and triterpenoid metabolism) enhanced in our study under non-stressed conditions (Jia et al., 2024a; Jia et al., 2024b). The mechanistic synergy observed between stress resilience and secondary metabolism activation underscores the dual benefit of La application: it not only stimulates the production of valuable bioactive compounds under standard cultivation but may also serve as a protective agent to sustain yield and medicinal quality in marginal lands or under abiotic stress challenges, such as salinity.

### 4.2 Dual role of lanthanum in *Glycyrrhiza uralensis*: root-specific accumulation coordinating biomass enhancement and targeted upregulation of secondary metabolite pathways

Regulatory genes in secondary metabolite biosynthetic pathways critically determine accumulation levels through their expression intensity. As an abiotic elicitor, La³^+^ ions trigger a coordinated response involving both primary and secondary metabolism. The elicitation effect manifests through enhanced organogenesis, particularly in root development, resulting in significantly increased biomass accumulation. Simultaneously, La treatment activates the expression of key biosynthetic genes in both the mevalonate and phenylpropanoid pathways, leading to the enhanced production of valuable secondary metabolites including glycyrrhizic acid, liquiritin, and their derivatives. In the glycyrrhizic acid/glycyrrhetinic acid pathway, SQE (Li et al., 2017)and CYP88D6 (Chiyo et al., 2024) are key genes whose elevated expression promotes accumulation. Our integrated metabolomic-transcriptomic analysis reveals that La treatment significantly upregulated SQE (r = 0.90, *p* < 0.05) and CYP88D6 (r = 0.97, *p* < 0.01) expression, concomitant with substantially increased glycyrrhizic acid content, demonstrating a strong positive correlation. This indicates that La-induced overexpression of SQE and CYP88D6 likely drives glycyrrhizic acid biosynthesis.

Similarly, in the liquiritin/liquiritigenin pathway, PAL (Ji et al., 2025), C4H (Wang et al., 2021), 4CL, and CHS play essential roles: PAL catalyzes phenylalanine to cinnamic acid, C4H converts cinnamic acid to p-coumaric acid, 4CL activates p-coumaric acid to p-coumaroyl-CoA, and CHS (with C4H) channels p-coumaroyl-CoA toward isoliquiritigenin—the precursor for liquiritin and liquiritigenin biosynthesis (Wang et al., 2023). La treatment markedly enhanced expression of PAL (r = 0.92, *p* < 0.05), C4H (r = 0.96, *p* < 0.01), 4CL (r = 0.93, *p* < 0.01), and CHS (r = 0.94, *p* < 0.01), correlating strongly with elevated liquiritigenin and isoliquiritigenin accumulation. These results demonstrate that La triggers coordinated upregulation of phenylpropanoid pathway genes, enhancing liquiritin/liquiritigenin production.

Concurrently, La treatment significantly increased *Glycyrrhiza uralensis* taproot length, biomass (Jia et al., 2024a), yield, and quality (Jia et al., 2024b). Treated plants exhibited substantially higher fresh weight (FW), dry weight (DW), and plant height than controls (*p* < 0.01, r > 0.6), aligning with the positive correlation between these growth parameters and major secondary metabolites. Our findings further demonstrate that La treatment significantly enhanced both root biomass and nutrient absorption capacity. The observed upregulation of key nutrient transporters, coupled with increased root surface area, synergistically improves the plant's ability to acquire essential minerals and precursors necessary for secondary metabolite biosynthesis. This enhanced nutrient uptake capacity directly supports the increased biosynthesis of phenylpropanoid and terpenoid compounds observed in La-treated plants. Crucially, as primary metabolites (directly linked to biomass) serve as precursors for secondary metabolism, and given the root-localized accumulation of glycyrrhizic acid, liquiritin, and liquiritigenin, we investigated La partitioning. ICP-MS quantification confirmed pronounced root-specific La accumulation (*p* < 0.001).

Root-accumulated La enhances precursor availability by increasing root biomass (FW/DW), while concurrently activating two pivotal biosynthetic pathways: (1) triterpenoid metabolism, through the induction of *SQE* and *CYP88D6* gene expression, enhancing glycyrrhizic acid biosynthesis; and (2) phenylpropanoid flux, via coordinated upregulation of *PAL*, *C4H*, *4CL*, and *CHS*, thereby promoting the production of liquiritin and liquiritigenin. To further elucidate how La stimulates metabolic flux into plastids—key sites for the synthesis of these specialized metabolites—we performed a targeted re-analysis of our transcriptome data to identify differentially expressed plastidial transporter genes. This analysis revealed significant upregulation of several transporters critical for precursor import into plastids. Specifically, the probable anion transporter 3, chloroplastic (PHT4;2), essential for inorganic phosphate import and energy homeostasis, was markedly upregulated (Log_2_FC = 1.4, *p* < 0.001). The Glucose-6-phosphate/phosphate translocator 2 (GPT2), which supplies carbon skeletons and NADPH for the shikimate and phenylpropanoid pathways, was also significantly enhanced (Log_2_FC = 2.3, *p* < 0.001). Moreover, the Triose phosphate/phosphate translocator (TPT), central to the distribution of photoassimilated carbon, showed increased expression (Log_2_FC = 1.1, *p* < 0.001). Although not a transporter, the strong upregulation of Beta-glucosidase 46 (BGLU46) (Log_2_FC = 1.3, *p* < 0.001) implies enhanced remobilization of glycoside-conjugated precursors (**Supplementary Table 6**). Collectively, the coordinated upregulation of these genes demonstrates that L not only boosts biomass but also specifically enhances the expression of transporters that facilitate substrate influx into plastids, thereby ensuring a sufficient supply of carbon, energy, and reducing equivalents required for the accelerated biosynthesis of glycyrrhizic acid and liquiritin. This dual mechanism, integrating increased precursor supply with transcriptional reprogramming of both biosynthetic and transporter genes, synergistically promotes the accumulation of pharmaceutically valuable metabolites in *Glycyrrhiza uralensis*.

### 4.3 Safety assessment of residual lanthanum in medicinal *Glycyrrhiza uralensis*

Rare earth elements (REEs), which are recognized as bioactive compounds with potential health benefits, have an estimated safe daily intake range of 6–60 mg for adults (Ferreira et al., 2022). Quantitative analysis of La in *Glycyrrhiza uralensis* after treatment showed a maximum residual concentration of 6.67 μg/g dry weight (**Supplementary Table 3**). This value constitutes less than 0.11% of the lower limit of the safe intake range and is consistent with residual levels reported in previous agricultural applications of REEs. Based on current experimental data, these findings indicate that the consumption of *Glycyrrhiza uralensis* treated with 100 mM La may pose a limited health risk.

### 4.4 Current limitations and future research priorities

While this study demonstrates La's dual mechanisms in enhancing *Glycyrrhiza uralensis* secondary metabolites—specifically glycyrrhizic acid, glycyrrhetinic acid, liquiritin, liquiritigenin, isoliquiritin, and isoliquiritigenin—via root biomass modulation and upregulation of key biosynthetic genes (CYP88D6, SQE, PAL, C4H, 4CL, CHS), its scope is confined to these six target compounds. Critical limitations necessitate future research focusing on: (1) incorporating comprehensive proximate analysis (e.g., protein, fiber, ash, and lipid content) to more fully evaluate the impact of La on biomass composition and nutritional quality; and (2) exploring the potential synergistic effects of integrating La application with other established biotechnological approaches, such as grafting onto resistant rootstocks, to further enhance stress resilience and metabolic yield in medicinal plants; (3) employing additional biochemical characterization techniques, such as spectrophotometric enzyme activity assays and isoelectric focusing, to provide deeper functional insights into the post-transcriptional and post-translational regulation of key biosynthetic enzymes in response to La treatment; and (4) directly investigating the application of these enhanced extracts in established pharmacological assays, including models for detoxification effects and studies on abnormal proliferation pathways such as *in vitro* toxicity or cancer cell line assays, to fully translate the agronomic advancement into potential therapeutic benefits.

## 5 Conclusion

In conclusion, foliar spray with 100 mM LaCl_3_ for seven consecutive days significantly enhances the accumulation of key secondary metabolites in *Glycyrrhiza uralensis*. This concentration was determined to be both effective and safe, as it substantially improves the yield and quality of medicinal herbs while remaining within the established safety thresholds for lanthanum application in agriculture (Ferreira et al., 2022). The proposed mechanism involves two synergistic actions: (1) increasing the pool of primary metabolic precursors serving as substrates for secondary metabolite biosynthesis, and (2) upregulating the expression of key biosynthetic genes (e.g., CYP88D6, SQE, PAL, C4H, 4CL, CHS) responsible for the synthesis of metabolites such as glycyrrhizic acid and liquiritin. This dual effect, ensuring both substrate availability and enhanced enzymatic capacity, ultimately promotes the targeted accumulation of pharmacologically relevant secondary compounds.

## Data Availability

The datasets presented in this study can be found in online repositories. The names of the repository/repositories and accession number(s) can be found in the article/**Supplementary Material**.
