# Supplementary material for: Lanthanum enhances biomass and bioactive metabolite production in Glycyrrhiza uralensis via coordinated gene regulation
Source: Front Plant Sci. 2025 Oct 16;16:1696545. doi: 10.3389/fpls.2025.1696545 (PMC12571737; doi:10.3389/fpls.2025.1696545)
Supplement: Supplementary file 2 [file DataSheet1.docx]

**Lanthanum Enhances Biomass and Bioactive Metabolite Production in *Glycyrrhiza uralensis* via Coordinated Gene Regulation**

Yuanyang Shao^1,2#^, Yushi Wang^1#^, Yunhao Zhu^3^, Chuanxin Liu^4^, Yashun Wang^1^, Xingyu Guo^1^, Enai Zhai^1^, Huiqin Zou^1*^, Yonghong Yan^1*^

1 School of Chinese Materia Medica, Beijing University of Chinese Medicine, Beijing, China;

2 Department of Chinese Medicine, The First Affiliated Hospital of Zhengzhou University, Zhengzhou, China;

3 School of Pharmacy, Henan University of Chinese Medicine, Zhengzhou, China;

4 Luoyang Key Laboratory of Clinical Multiomics and Translational Medicine, Key Laboratory of Hereditary Rare Diseases of Health Commission of Henan Province, Henan Key Laboratory of Rare Diseases, Endocrinology and Metabolism Center, The First Affiliated Hospital, and College of Clinical Medicine of Henan University of Science and Technology, Luoyang, China.

Supplementary Table 1 qRT-PCR primer sequences

| Accession Name | Primer | Sequence |
| --- | --- | --- |
| Actin-F | Forward Primer | 5’-TCTGGATCTTGCTGGCCGTGAT-3’ |
| Actin-R | Reverse Primer | 5’-TGGGCAACGGAATCTCTCAGCT-3’ |
| SQE-F | Forward Primer | 5’-AATATGAGGAGAACTCCGTGAAAG-3’ |
| SQE-R | Reverse Primer | 5’-GCCATGATCTGGGGAATAGC-3’ |
| CYP88D6-F | Forward Primer | 5’-TGCATGTCCGCTGCCACTTTGT-3’ |
| CYP88D6-R | Reverse Primer | 5’-TCGCCGATAAGAGGCCATCCCA-3’ |
| C4H-F | Forward Primer | 5’-AGAGCACCAGCAACGAAGGACT-3’ |
| C4H-R | Reverse Primer | 5’-TGGGTGGTTCACAAGCTCAGCA-3’ |
| 4CL-F | Forward Primer | 5’-TCCGATGTTGCTGTGGTCCCAA-3’ |
| 4CL-R | Reverse Primer | 5’-TTTGGAAGACCCGCTGCAAGCT-3’ |
| CHS-F | Forward Primer | 5’-ACGTGCTGAGCGAGTATGGGAA-3’ |
| CHS-F | Reverse Primer | 5’-AGCCTGCAAGGGAACACTGTGA-3’ |
| PAL-F | Forward Primer | 5’-CCTTGCAGTGCCACGTACCCAT-3’ |
| PAL-R | Reverse Primer | 5’-ACCCTCGCACCTTCCACCTCTT-3’ |

Supplementary Table 2 Content of six major secondary metabolites in *Glycyrrhiza uralensis*

| Identification | Formula (mol ion) | t_R_ (min) | Concentrations (μg/g, La) | Concentrations (μg/g,W) |
| --- | --- | --- | --- | --- |
| Glycyrrhizic acid | C_42_H_62_O_16_ | 6.12 | 268.72 ± 16.89 | 179.77 ± 11.53 |
| Glycyrrhetinic acid | C_30_H_46_O_4_ | 9.23 | 0.034±0.0043 | 0.025±0.0041 |
| Liquiritin | C_21_H_22_O_9_ | 3.73 | 11.30 ± 0.95 | 5.00 ± 2.57 |
| Liquiritigenin | C_15_H_12_O_4_ | 4.89 | 165.38 ± 19.24 | 28.39 ± 7.03 |
| Isoliquiritin | C_21_H_22_O_9_ | 4.43 | 104.08±9.41 | 94.24±2.56 |
| Isoliquiritigenin | C_15_H_12_O_4_ | 5.92 | 84.33±3.49 | 77.00±4.93 |

Supplementary Table 3 L content in roots and leaves of *Glycyrrhiza uralensis*

| Sample ID | Sample weight (g) | Final volume (mL) | Element analyzed | Concentration in solution (mg·L⁻¹) | Dilution factor | Element content (mg·kg⁻¹) |
| --- | --- | --- | --- | --- | --- | --- |
| Root1 | 0.0236 | 10 | La | 0.6672 | 1 | 282.73 |
| Root2 | 0.0206 | 10 | La | 0.6095 | 1 | 295.88 |
| Root3 | 0.0251 | 10 | La | 0.6814 | 1 | 271.46 |
| Leaf1 | 0.0485 | 10 | La | 0.0502 | 1 | 10.36 |
| Leaf2 | 0.0308 | 10 | La | 0.1097 | 1 | 35.61 |
| Leaf3 | 0.0361 | 10 | La | 0.0734 | 1 | 20.34 |

Supplementary Table 4 RNA integrity and purity parameters of *Glycyrrhiza uralensis* samples prior to library construction

| Sample | Total (µg) | OD_260/280_ | OD_260/230_ | 25S/18S | RIN |
| --- | --- | --- | --- | --- | --- |
| La1 | 1.37 | 2.17 | 0.71 | 0.80 | 6.80 |
| La2 | 1.4 | 2.12 | 0.68 | 0.80 | 6.80 |
| La3 | 1.26 | 2.13 | 0.67 | 0.90 | 7.00 |
| W1 | 2.07 | 2.11 | 1.08 | 1.20 | 6.80 |
| W2 | 1.12 | 2.10 | 0.78 | 1.30 | 7.50 |
| W3 | 1.47 | 2.17 | 0.97 | 0.90 | 7.00 |


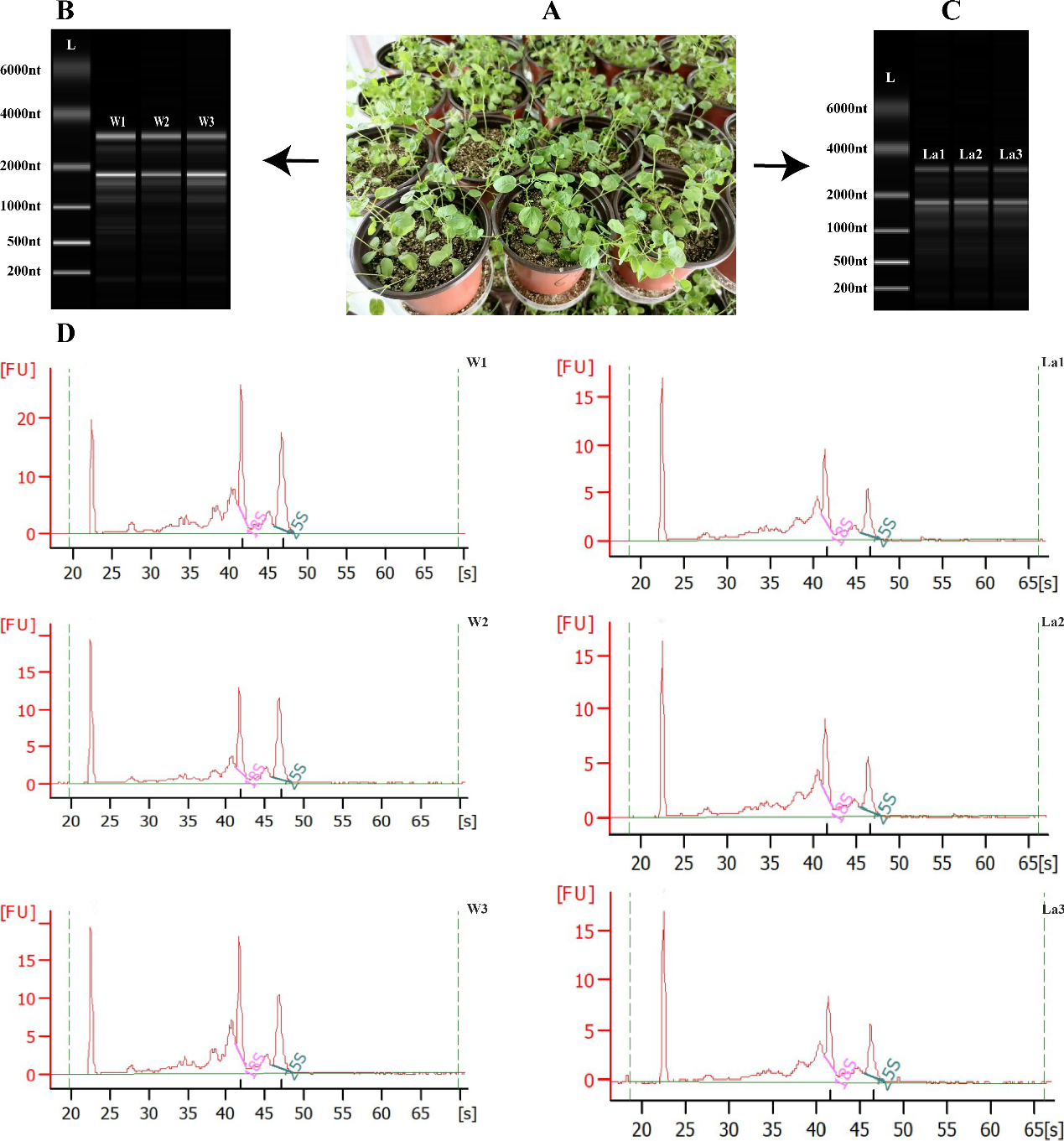


Supplementary Figure 1 Quality assessment of total RNA from GU seedlings under W and La.**(A)** Glycyrrhiza seedlings; **(B)** Agarose gel electrophoresis of W group total RNA; **(C)** Agarose gel electrophoresis of La group total RNA (Lane M: RNA markers); **(D)** HPLC profile of total RNA.

Supplementary Table 5 Summary of RNA sequencing data quality and mapping statistics for *Glycyrrhiza uralensis* samples

| Sample | Total Raw Reads (M) | Total Clean Reads (M) | Clean Reads Ratio (%) | Clean Reads Q20(%) | Clean Reads Q30(%) | Total Mapping (%) | Uniquely Mapping (%) |
| --- | --- | --- | --- | --- | --- | --- | --- |
| La1 | 45.47 | 44.58 | 98.04 | 98.03 | 94.22 | 91.90 | 89.42 |
| La2 | 42.56 | 41.69 | 97.96 | 97.89 | 93.91 | 91.14 | 88.71 |
| La3 | 44.80 | 43.86 | 97.90 | 97.97 | 94.08 | 92.07 | 89.59 |
| W1 | 42.73 | 41.42 | 96.93 | 98.1 | 94.47 | 91.80 | 89.20 |
| W2 | 45.63 | 44.35 | 97.19 | 98.1 | 94.34 | 92.23 | 89.62 |
| W3 | 45.67 | 44.62 | 97.70 | 98.06 | 94.3 | 91.67 | 89.13 |
